# Oxidative and anti-oxidative status in muscle of young rats in response to six protein diets

**DOI:** 10.1038/s41598-017-11834-5

**Published:** 2017-10-13

**Authors:** Jing Zhu, Xiao Li, Hao Qi, Zetong Gu, Shangxin Song, Xiangli Yang, Guanghong Zhou, Chunbao Li

**Affiliations:** 1 Key Laboratory of Meat Processing and Quality Control, MOE, Key Laboratory of Meat Processing, MOA, Jiangsu Synergetic Innovation Center of Meat Processing and Quality Control, College of Food Science and Technology, Nanjing Agricultural University, Nanjing, 210095 P.R. China; 2 Jiangsu Key Laboratory for Information Agriculture, National Engineering and Technology Center for Information Agriculture, College of Agriculture, Nanjing Agricultural University, Nanjing, 210095 P.R. China; 3 Department of Electrical and Computer Engineering, Northeastern University, Boston, MA 02115 USA; 4 School of Food Science, Nanjing Xiaozhuang University, Nanjing, 211171 P.R. China; 5 Department of Molecular and Human Genetics, Baylor College of Medicine, Houston, TX 77030 USA

## Abstract

We investigated the impact of six protein diets on oxidation and anti-oxidation status in the muscle of young rats. Rats were fed six protein diets for 14 days, including casein (control), and proteins isolated from soy, fish, chicken, pork and beef. Grx1, Trx1 and other oxidative metabolic indices in muscle were quantified. Compared with the casein diet, the soy protein diet had a similar oxidation level, but higher GSH and lower SOD activities. The chicken and fish protein groups had lower GSH and higher SOD activities, the pork protein group showed lower Grx1 levels than the casein group and the beef protein group showed the highest GSH, Grx1 and Trx1 levels as reflected by RT-PCR, Western blotting and immunohistochemistry analyses. Intake of meat proteins showed higher ROS and T-AOC but lower MDA levels than non-meat proteins, which may be due to the increase in Grx1 and Trx1 expression and other antioxidants. Meat proteins are more conducive to muscle of growing rats.

## Introduction

Contracted skeletal muscle produces reactive oxygen species (ROS), which brings about muscle performance interference, including muscle lassitude and oxidative stress^1^. ROS is a class of highly reactive free radicals or molecules containing oxygen, including superoxides, peroxides, hydroxyl radicals and singlet oxygen^2^. High ROS level may have a substantial influence on cell structures which further leads to a significant damage. ROS plays an important role in cell signaling^3^, homeostasis^4^ and Parkinson’s disease^5^. ROS production is inevitable, and thus the cells have anti-oxidation defense system to control the ROS production^6^. The total antioxidant capacity (T-AOC) includes enzymatic and non-enzymatic antioxidants in cellular and extracellular environments^7^. One of the vital enzymatic antioxidants in skeletal muscle is superoxide dismutase (SOD). It can convert ROS to hydrogen peroxides, and then other enzymes convert hydrogen peroxides to water^8^. The reduced SOD activity would promote lipid peroxidation to produce malondialdehyde (MDA) in the presence of metal ions. MDA is toxic to the cells, which could be coupled with protein molecules and induce apoptosis^9^. In addition, glutaredoxin (Grx) and thioredoxin (Trx) also belong to the antioxidant systems that are different from the above enzymes. Grx and Trx are small peptides encoded by genes Grx1 and Trx1 respectively^10^. Cancer cells can survive and proliferate under high concentrations of ROS because they can produce elevated levels of Grx and Trx that have the ability to remove ROS^11–13^. Grx catalyzes the reduction of disulfides via reduced glutathione (GSH)^10^. There is an active site, disulfide bond in Grx1. It can be reduced or oxidized depending on the cellular level of ROS^11^. Trx scavenges ROS and reduces disulfide bonds to maintain redox homeostasis with thioredoxin reductase and NADPH^12^.

Diet intake promotes a major oxidative response at the cellular level, and further alters the metabolic status of an organ including muscles^14^. Many previous studies have focused the induction of high-fat diets to metabolic dysfunction by oxidative stress in skeletal muscles^15–17^. Recent studies focused more on dietary supplements for antioxidant use in skeletal muscles^18–22^. Much concern has been taken about the effect of high-protein diets on obesity^23–25^ and muscle strength enhancement^26–28^. However, little is known about the impact of dietary proteins from different sources on the oxidative status of muscles at a recommended intake. Intake of different dietary proteins may result in the difference in anti-oxidative status *in vivo*.

To explore how dietary proteins of different sources affect the oxidative status and its regulation in muscle, we fed young male rats for 7 and 14 days with six protein diets. The six proteins were casein and proteins from soy, fish, chicken, pork and beef. We, for the first time, to evaluate the effect of different diet proteins at a standard intake of 20% on oxidative status and antioxidant activities in rat muscle. To get robust comparisons, we set the casein diet as control, and the diet impact was compared on the basis of the ratios of the soy and meat proteins to the control.

## Results

### Diets had different impacts on oxidative status in rats muscle

**Figure 1:**
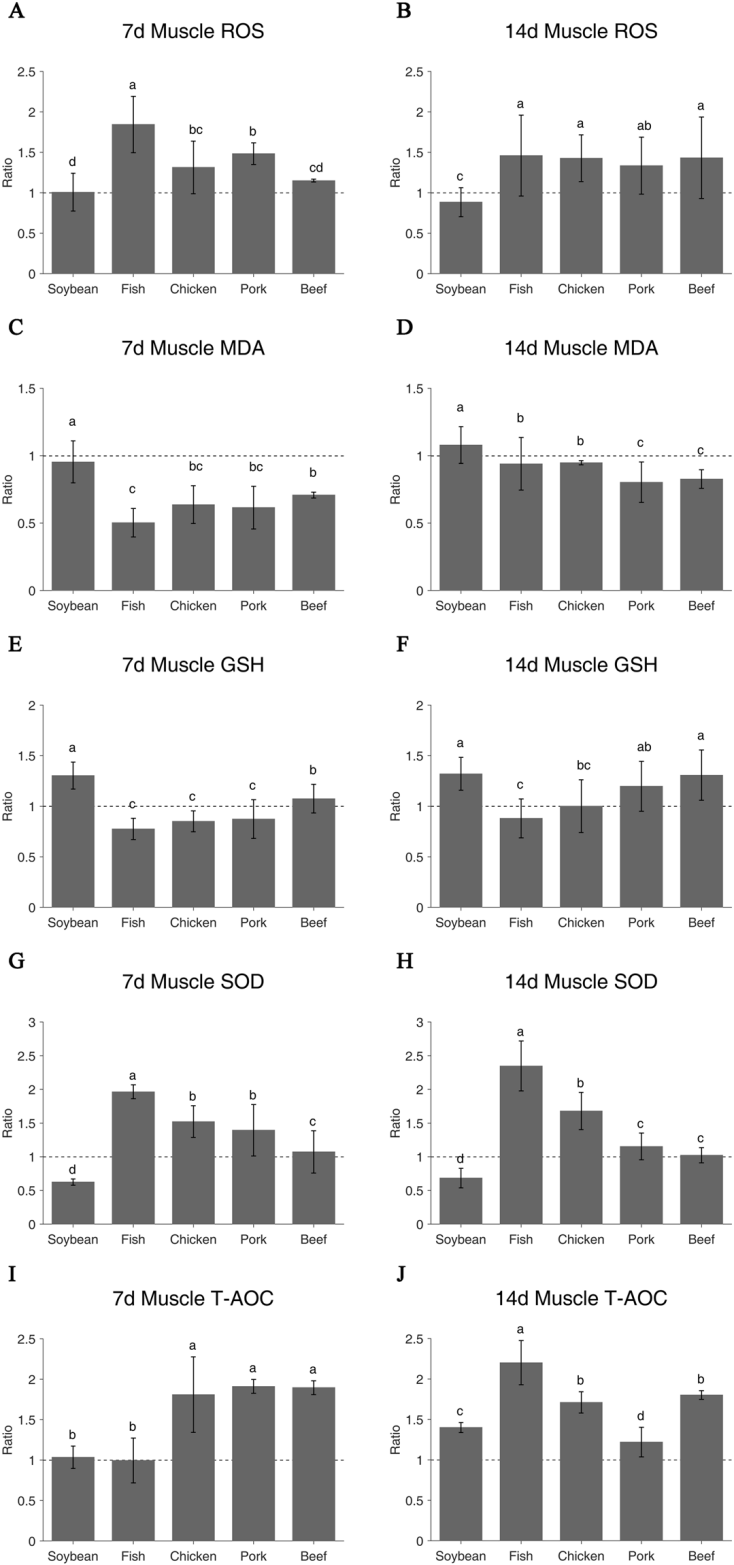
Oxidative status of different protein diets in muscle on day 7 and day 14. ROS (**A**,**B**), MDA (**C**,**D**), GSH (**E**,**F**), SOD (**G**,**H**) and T-AOC (**I**,**J**). Ratio of relative expression = Treatment group/Control group. Casein group is the control and the value is 1. The a/b/c/d indicates significant difference (P < 0.05).

On day 7 of feeding, the soy protein group as same as casein had significantly lower ROS level but higher MDA level than fish, chicken and pork protein groups (P < 0.05, Fig. 1A and C). Among four meat protein groups, the fish protein group showed the highest ROS value and the beef protein group was the lowest (P < 0.05, Fig. 1A). However, the opposite results were observed for the MDA values (P < 0.05, Fig. 1C). On day 14, all meat protein diets showed higher ROS level than the casein and soy protein groups (P < 0.05, Fig. 1B). However, all meat protein groups, in particular to pork and beef protein groups, had lower MDA levels (P < 0.05, Fig. 1D). The highest MDA value was observed for the soy protein group but the lowest was the pork protein group (P < 0.05, Table 1).

**Table 1:** Oxidation metabolic indices of six protein diets in muscle on day 7 and day 14.

Protein diets	Casein	Soybean	Fish	Chicken	Pork	Beef	period
ROS	48754 ± 18376	49151 ± 11391	89927 ± 16965	64066 ± 15843	72347 ± 6514	56104 ± 851	7d
F/mg pro	46758 ± 14353	41330 ± 8391	68258 ± 23394	66701 ± 13512	62459 ± 16459	67025 ± 23591	14d
MDA	4.743 ± 0.597	4.524 ± 0.742	2.387 ± 0.501	3.021 ± 0.663	2.915 ± 0.750	3.360 ± 0.105	7d
nmol/mg pro	3.414 ± 0.632	3.685 ± 0.463	3.209 ± 0.664	3.236 ± 0.055	2.743 ± 0.512	2.822 ± 0.236	14d
GSH	3.943 ± 0.082	5.140 ± 0.525	3.057 ± 0.413	3.356 ± 0.407	3.443 ± 0.755	4.240 ± 0.556	7d
μmol/g pro	4.964 ± 0.392	6.555 ± 0.811	4.368 ± 0.951	4.971 ± 1.303	5.947 ± 1.225	6.493 ± 1.236	14d
SOD	2.600 ± 0.259	1.623 ± 0.121	5.111 ± 0.268	3.957 ± 0.611	3.624 ± 0.993	2.788 ± 0.817	7d
U/mg pro	2.245 ± 0.187	1.535 ± 0.325	5.271 ± 0.831	3.768 ± 0.619	2.587 ± 0.444	2.296 ± 0.253	14d
T-AOC	0.141 ± 0.032	0.146 ± 0.020	0.141 ± 0.039	0.256 ± 0.066	0.270 ± 0.012	0.268 ± 0.012	7d
/mg pro	0.184 ± 0.027	0.258 ± 0.012	0.406 ± 0.050	0.315 ± 0.024	0.225 ± 0.034	0.332 ± 0.010	14d

Values are means ± SE; n = 8 per group.

### Diets altered the antioxidant activities of GSH, SOD and T-AOC

After seven-day feeding, the soy protein group had higher GSH level than the casein and four meat protein groups (P < 0.05, Fig. 1E). The beef protein group showed the higher GSH activity than the four meat protein groups (P < 0.05). The 14-day GSH activities were in accordance with the 7-days results (Fig. 1F). On the contrary, the soy protein group showed the lowest SOD activity (P < 0.05, Fig. 1G) and among four meat protein groups, the SOD values were ranked as follows: fish > chicken and pork > beef (P < 0.05). The 14-day SOD results were consistent with the 7-day results (Fig. 1H). In terms of T-AOC, the soy and fish protein groups had the same T-AOC as the casein group (P > 0.05), while the other groups had much stronger T-AOC (P < 0.05, Fig. 1I). On day 14, T-AOC increased greatly in the soy and fish protein groups, but decreased in the pork protein group (Fig. 1J).

### Grx1 and Trx1 mRNA showed significant responses to diets

**Figure 2:**
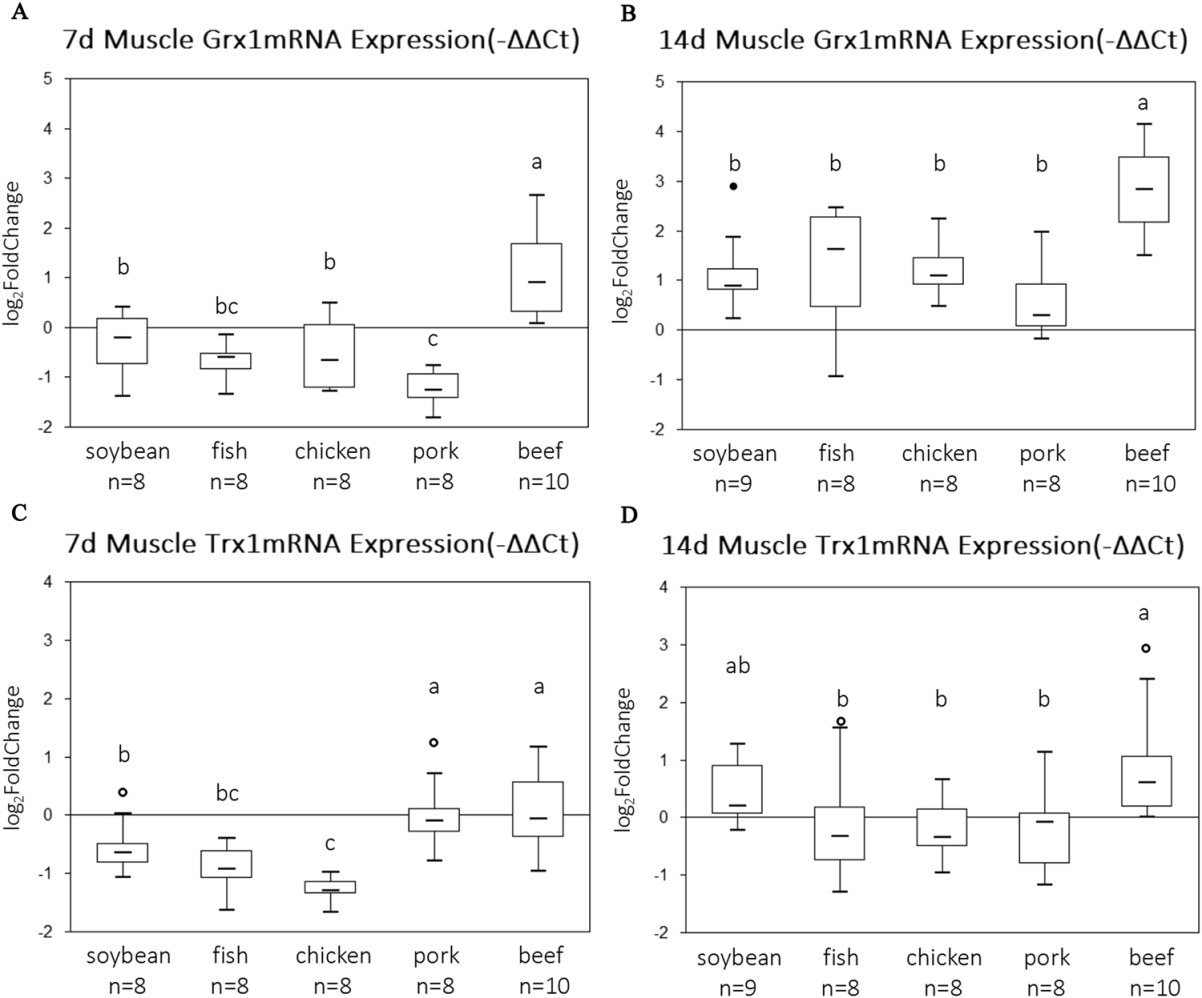
The qRT-PCR data of Grx1 (**A**,**B**) and Trx1 (**C**,**D**) mRNA expression in rats muscle. The boxes represent the 25^th^ through 75^th^ percentiles, the horizontal lines represent the medians. The distance from the 25^th^ to the 75^th^ percentiles is the interquartile range (IQR). The hollow dot represents the outlier between 1.5 times and 3 times of IQR far away from 25^th^ or 75^th^ percentiles. The whiskers represent the minimum and maximum values except outliers. Casein group is the control and the value is 0. The a/b/c indicates significant difference (P < 0.05).

On the mRNA level, the beef protein diet had the highest Grx1 value on day 7, while the lowest value was for the pork protein group (P < 0.05, Fig. 2A). On the day 14 of feeding, the highest Grx1 mRNA level was still for the beef protein group (P < 0.05, Fig. 2B), and there was no significant difference between any other diet groups (P > 0.05). The Trx1 mRNA levels in the pork and beef protein groups were the highest on day 7, but the lowest was for the chicken protein group (P < 0.05, Fig. 2C). On day 14, the highest value was still for the beef protein group (P < 0.05, Fig. 2D).

### Grx1 and Trx1 proteins also showed significant responses

**Figure 3:**
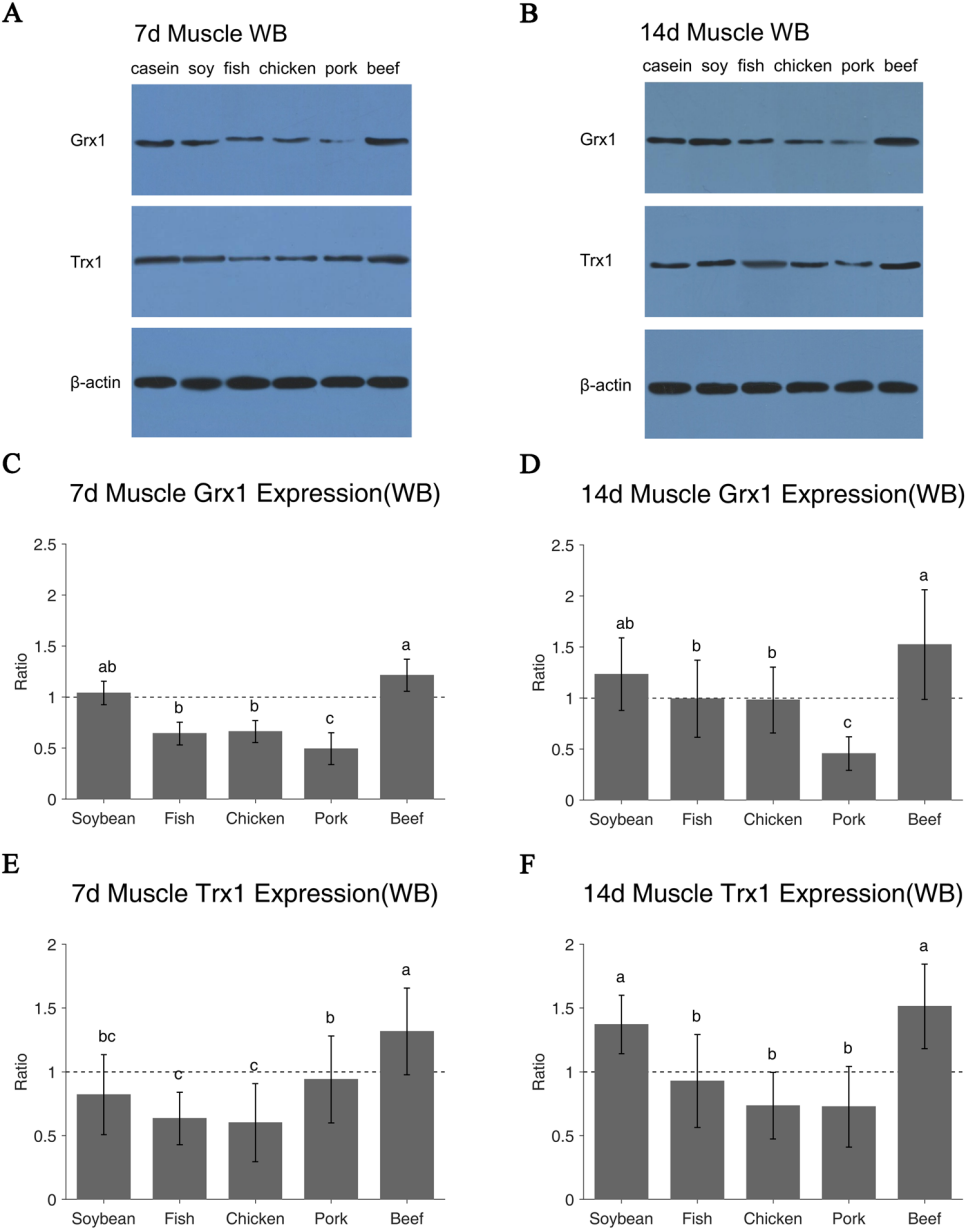
Grx1 and Trx1 protein expression of different protein diets in rats muscle on day 7 and day 14 were examined using Western blotting (**A**,**B**). β-Actin was used as reference. Relative expression of target protein = [Target protein (OD)/Reference protein(OD)] * 10, Ratio of relative expression = Treatment/Control. Casein group is control as 1 (**C**–**F**). Values are means ± SE; n = 8–10 per group. The a/b/c indicates significant difference (P < 0.05).

Western-blotting (WB) results (Fig. 3A and B) confirmed the RT-PCR results. The beef protein diet had higher Grx1 protein level than the casein and soy protein diets on day 7, while the intake of fish, chicken and pork proteins decreased the Grx1 levels (P < 0.05, Fig. 3C). On day 14, the Grx1 protein levels of all diets increased except that of the pork protein group. The beef protein group still showed the highest value but the pork protein group’s value was the lowest (P < 0.05, Fig. 3D). Intake of beef protein diet for 7 days resulted in the highest Trx1 protein level, but the soy, fish and chicken groups showed lower values as compared with the casein group (P < 0.05, Fig. 3E). After 14-day feeding, all diets increased the Trx1 protein levels except the pork protein group. The soy and beef protein groups showed the highest Trx1 protein expression (P < 0.05, Fig. 3F). There was no significant difference among fish, chicken and pork groups (P > 0.05, Fig. 3F).

### Immunohistochemical staining results of Grx1 and Trx1

**Figure 4:**
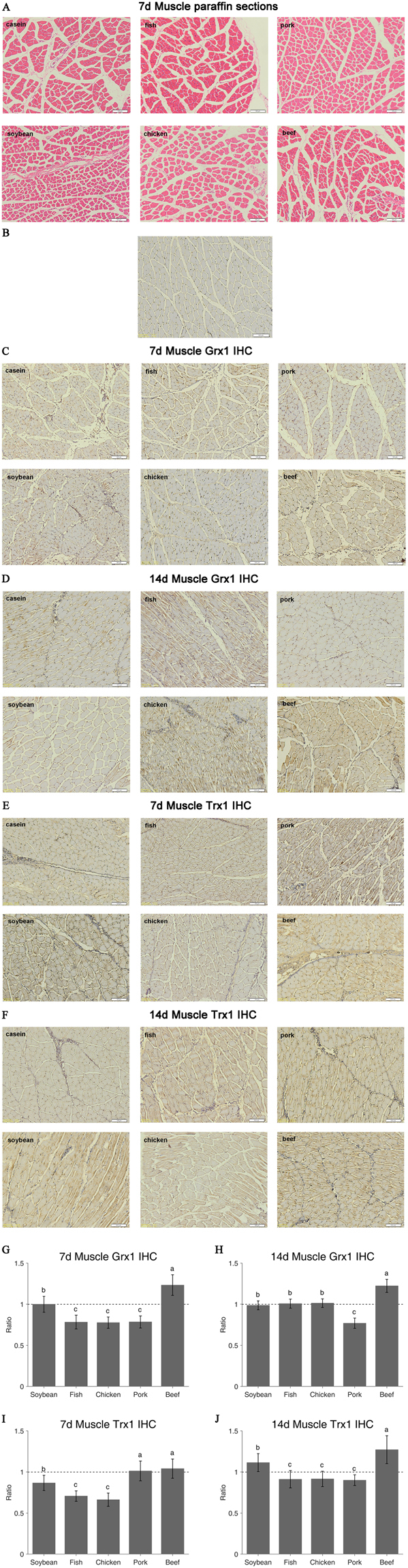
Paraffin sections of muscle from six diets for IHC (**A**, original magnification, *100); IHC staining of Grx1 and Trx1 in muscle on day 7 and day 14 are shown, including the negative slide without positive signal (**B**). The positive stains were yellow or brown signals and nuclei were stained blue with hematoxylin (**C**–**F**) and the OD of the yellow area was measured as semi-quantitative results (**G**–**J**). Ratio of relative expression = Treatment group/Control group. Casein group is the control and the value is 1. Values are means ± SE, n = 6 * 3 per group. The a/b/c indicates significant difference (P < 0.05).

Immunohistochemical (IHC) staining (Table 2) results further confirmed the results of RT-PCR and western blotting. The paraffin sections of each group indicated good quality of muscle tissues for IHC (Fig. 4A). Grx1 was observed in the muscle fibers (brownish yellow granules, Fig. 4C–F), but did not exist in negative control (Fig. 4B). Compared to the casein and soy protein groups, intake of beef protein diet induced stronger Grx1 staining, while weaker signals were observed in the fish, chicken and pork protein groups (P < 0.05, Fig. 4G). The pork and beef protein diets caused similar optical density (OD) values of Trx1 to the casein group (P > 0.05, Fig. 4I), while the soy, fish and chicken protein groups had lower OD values (P < 0.05, Fig. 4I). After 14-day feeding, the pork protein group had the lowest Grx1 IHC value (P < 0.05, Fig. 4H), while the beef protein group still had the highest OD values of Grx1 and Trx1 (P < 0.05, Fig. 4H and J). The white protein diets, fish and chicken, showed similar Grx1 and Trx1 IHC values (P > 0.05). Trx1 IHC value of the pork protein diet did not differ from the chicken and fish meat protein groups on day 14 (P > 0.05, Fig. 4J).

**Table 2:** Grx1 and Trx1 IHC relative expression of six protein diets in rats muscle.

Protein diets	Casein	Soybean	Fish	Chicken	Pork	Beef	period
Grx1	3.046 ± 0.261	3.046 ± 0.295	2.386 ± 0.253	2.364 ± 0.208	2.391 ± 0.224	3.757 ± 0.382	7d
OD	3.086 ± 0.202	3.047 ± 0.167	3.111 ± 0.171	3.130 ± 0.159	2.376 ± 0.191	3.779 ± 0.243	14d
Trx1	3.718 ± 0.329	3.225 ± 0.345	2.628 ± 0.236	2.464 ± 0.297	3.765 ± 0.446	3.870 ± 0.434	7d
OD	3.215 ± 0.170	3.581 ± 0.348	2.929 ± 0.338	2.944 ± 0.300	2.895 ± 0.210	4.088 ± 0.546	14d

IHC relative expression of target protein = [Target protein(OD)] * 10, Values are means ± SE; n = 6 * 3 per group.

## Discussion

Intake of protein plays a certain role in the maintenance of metabolism balance in muscle^29,30^. During this process, ROS is produced. ROS accumulation would activate the enzymatic and non-enzymatic antioxidant responses^31^, including SOD^8^, GSH^32^ and antioxidant protein, Grx1 and Trx1^10^. The total antioxidant system reduces ROS level and MDA generation. The T-AOC reflects a co-ordinate effect of the above antioxidant systems^33^.

Previous study showed that intake of meat proteins induced higher ROS level in rat muscle than the intake of casein and soy protein did. Meanwhile, SOD and T-AOC values were also higher in meat protein groups. One possible explanation for this is meat protein intake enhances muscle metabolism compared to intake of non-meat proteins. Previous research indicated that ROS may induce beneficial metabolic adaptations^34^. Recent studies indicated that ROS had a complex relationship with aging. Increasing ROS level in mitochondria can promote longevity, but high levels of ROS are toxic^35^. Contrary to ROS, as a marker of oxidative stress^36^ and oxidation end-product^37^, MDA was reduced by intake of meat proteins. It may be because meat proteins showed higher SOD levels than the casein and soy protein. Cells possess many pathways for neutralizing ROS, including a variety of SOD. Mice lacking SOD have high levels of oxidative damage in many tissues including skeletal muscle^38^. The SOD mRNA levels remained relatively stable in heat-stressed cells^39^, which may manifest that SOD results on day 14 of feeding were consistent with those of day 7 feeding. The fish protein group had the highest SOD level that may make a great contribution to T-AOC left in muscle. The beef and pork protein groups had higher GSH levels to protect against oxidative stress. GSH is a co-factor of several detoxifying enzymes and scavenges hydroxyl radical and singlet oxygen directly to reduce MDA^40^.

Meanwhile, Grxs play key roles in cellular redox regulation. Grx1 is diminished in the intermembrane space of mitochondria from aged heart^41^. Grx1 is also related to GSH, which glutaredoxins utilize the reducing power of GSH to maintain and regulate the cellular redox state and redox-dependent signaling pathways^42^. The fish and chicken protein groups had similar Grx1 levels based on western blotting and IHC results on day 14 of feeding since they have the similar GSH levels. On the other hand, Trxs have a remarkable number of functions in mammalian cells^43^ and are maintained independently from the GSH system. Moreover, Trx1 is required for the growth of the normal cells, while the Grx1 is not required^44^. The pork protein group had lower Grx1 and Trx1 levels than the casein group on the western blotting and IHC levels, which may cause lower T-AOC left in skeletal muscle. T-AOC could be useful to evaluate nutritional interventions for disease risk and prevention, including anti-aging strategies^45^. The beef protein group had much higher Grx1 and Trx1 expression and stronger T-AOC than the casein group. There may be a relevance between antioxidant indices and T-AOC values, but the measurement of T-AOC is considered as the cumulative effect of all the antioxidants in body, thus providing an integrated parameter rather than the simple sum of measurable antioxidants^33^. The 14-day intake had stronger gene expression and higher oxidation level than the 7-day intake, which indicates that a variety of dietary proteins are needed to avoid the damage caused by the long-term intake of a single dietary protein.

High quality diet protein is important for muscle growth^46,47^. Considering protein resources and costs, dietary protein supplements are always milk or plant proteins^48,49^, while animal protein would bring greater quantities of high-quality protein to people in developing countries^50^. Animal proteins, especially those from diets, seem to support better muscle protein synthesis than plant proteins^51^. The contents of leucine, isoleucine, phenylalanine + tyrosine, valine and lysine in soybean protein were significantly lower than those in these meat proteins^52^. The soy protein and casein diets for vegetarians showed no significant advantages for muscle health. As white meat, the fish and chicken protein groups showed similar results of oxidation and anti-oxitation in muscle because they had similar amino acid composition. And the chicken protein diet showed stable oxidative and anti-oxidative status from day 7 to day 14, which may be related the comprehensive and rich composition of amino acids according to amino acid score in previous publication^52^. However, beef protein was quite different from pork protein since they had different amino acid composition. The contents of leucine, lysine, isoleucine, phenylalanine + tyrosine, valine and serine in pork protein were significantly lower than those in beef protein. Previous studies showed that certain amino acids, including leucine, arginine and lysine, may play a critical role in optimizing efficiency of metabolic transformation to enhance muscle growth^53^. High dose of leucine may stimulate muscle protein synthesis and inhibit protein degradation in skeletal muscle^54^. Dietary l-arginine supplementation can increase muscle gain in growing-finishing pigs^55^. Lysine deficiency has been reported to reduce muscle mass^56^ and the activities of SOD in mice^57^. L-lysine treatment can enhance antioxidant activity by inhibiting the release of the inflammatory cytokine IL-6, which may involve upregulation of anti-inflammatory factors and subsequent downregulation of IL6. The fish protein group had the highest SOD activity, which could be due to the highest lysine concentration in the diet^52^.

In summary, we, for the first time, found that dietary meat proteins caused higher ROS production in young rat muscle than non-meat proteins and also higher antioxidant levels by increasing SOD activities, especially fish protein diet. As a result, the meat proteins had higher T-AOC but lower MDA levels. Meat proteins are more conducive to muscle of growing rats. The rats of beef protein diet group had the highest levels of Trx1 and Grx1 according to the RT-PCR, western blotting and IHC results, which is related to the highest GSH value of beef protein diet group. Nutritional benefits of mixtures of complementary protein sources are needed to avoid the damage caused by the long-term intake of a single dietary protein. The differences in oxidative status and antioxidant capacity may be related to the composition of amino acids in dietary proteins. Further work will be done to explore the underlying mechanism on how certain amino acids in diets regulate the oxidative status in muscle.

## Materials and Methods

### Diets

Diet were prepared by Jiangsu Xietong, Inc. (Nanjing, China) according to the formulation of AIN-93G diet^58^. The control diet was prepared by casein and the other five diets were made by replacing casein with purified proteins from soy, fish, chicken, pork and beef. The protein isolation was performed as previously described^52^.

### Animals and sample collection

One hundred and twenty three-week-old male Sprague Dawley rats were bought from Shanghai Laboratory Animal Research Center (Shanghai, China) and housed in pairs at the Animal Center of Nanjing Agricultural University. All rats were kept in a pathogen-free environment. The procedures for care and use of animals were approved by the Ethics Committee of Nanjing Agricultural University [License No. SYXK 2011–0037] and all applicable institutional and governmental regulations concerning the ethical use of animals were followed. All rats were fed one-week control diet, and then randomly assigned to six protein diet groups (20 rats per group), i.e., casein diet (control), soy protein diet, fish protein diet, chicken protein diet, pork protein diet and beef protein diet. On day 7 and day 14, 10 rats from each diet group were decapitated and thigh muscles of posterior limb were collected, quick-frozen in liquid nitrogen and then stored at −80 °C.

### Oxidation metabolic indices

Protein was quantified by the BCA Kit (A045-3). The oxidative status and antioxidant activities were quantified by ROS Kit (DCFH-DA, E004), MDA Kit (A003-1), T-AOC Kit (A015), GSH Kit (A006-2) and SOD Kit (A001-3), which were all from Nanjing Jiancheng Bioengineering Institute (Nanjing, China). In brief, muscle sample (0.1 g) was homogenized in 0.9 ml saline at 4 °C, centrifuged at 2500 rpm for 10 min, kept the total protein supernatant at 4 °C. The absorbance at 562 nm was recorded by an Mze Microplate Spectrophotometer (MD, USA) for the total protein concentration (BCA Kit). For ROS, muscle sample (0.1 g) was homogenized in 1.9 ml, centrifuged at 1000 g for 10 min and the supernatant was kept. Protein concentration of the supernatant was quantified by the BCA Kit. A 190 μl of supernatant was mixed with 10 μl 1.0 mmol/L DCFH-DA working solution, and then incubated at 37 °C for 30 min. The fluorescence intensity was detected under excitation wavelength at 485 nm and emission wavelength at 525 nm. A blank control was used as PBS with working solution. The results were recorded as fluorescence intensity per milligram protein. For MDA, 50 μl 1% total protein supernatant was mixed with 1 ml working buffer, and incubated at 95 °C for 50 min. The reaction mixture was cooled down by running water and centrifuged at 4000 rpm for 10 min. The absorbance of the supernatant (200 μl) was measured at 532 nm. The blank control was 100% ethanol and the standard control was 10 nmol/ml reference solution. GSH was recorded as the absorbance at 405 nm. A blank control was used that did not contain sample, and a standard control was used that contained 20 μmol/L GSH. For SOD, 20 μl 10% total protein was mixed with 220 μl working solution, and incubated at 37 °C for 20 min, and then the absorbance was recorded at 450 nm. The value of T-AOC was measured as the absorbance at 520 nm. Double distilled water was used for a blank control.

### RNA isolation and quantitative RT-PCR

Total RNA was isolated from muscles using the TAKARA MiniBEST Universal RNA Extraction Kit (No. 9767). The RNA concentration and quality were determined at 230 nm, 260 nm and 280 nm with a Nanodrop ND-2000 spectrophotometer (Thermo, Scientific, Waltham, MA). The samples were considered acceptable if A260/A280 and A260/A230 ratios were not smaller than 2.0. Then RNAs were reversely transcribed into cDNA using TAKARA PrimeScript Master Mix Kit (No. RR036A). All cDNAs were diluted to a minimum concentration of 111 ng/μl to serve as templates for subsequent PCR using the primers (Table 3). The quantitative melting curve of the target gene had one single peak, indicating that there was no nonspecific amplification and primer dimer formation in the amplification process^59^. RT-PCR was performed in QuantStudio™ 6 Flex Real-Time PCR System (Applied Biosystems, Foster, CA) with a total volume of 20 μl per well including 2 μl of the cDNA template and 18 μl of the TAKARA Master Mix Kit (No. RR420A). The results were normalized to the −ΔΔCT values of the casein group and a positive value indicated up-regulation on the mRNA level. The reference gene was β-actin. Four replicates were performed for each sample.

**Table 3:** Primers for qRT-PCR.

Gene	Primer sequence	Length
Trx-1	Sense: 5′AGCCCTTCTTTCATTCCCTCTG3′	150 bp
(target)	Anti-Sense: 5′ACTCCCCAACCTTTTGACCCTT3′	
Grx-1	Sense: 5′TCAGTCTGGAAAGGTGGTCG3′	147 bp
(target)	Anti-Sense: 5′TCTTGAATCGCATTGGTGTTG3′	
β-actin	Sense: 5′CCCATCTATGAGGGTTACGC3′	150 bp ordered
(reference)	Anti-Sense: 5′TTTAATGTCACGCACGATTTC3′	

Primers were synthesized by the Shanghai Sangon Biotech Company (Shanghai, China). Primer-specific detection tools:http://www.ncbi.nlm.nih.gov/tools/primer-blast/.

### Western Blotting

Muscle protein was extracted in chilled tissue protein extraction reagent (No. 78510, Thermo Pierce) with protease and phosphatase inhibitor cocktail (No. 78440, Thermo Pierce). Protein concentration was quantified by a BCA assay kit (No. P0010, Beyotime Biotech) and diluted to 10 μg/μl. Diluted protein mixture was mixed with an equal volume of loading buffer (Solarbio, Beijing, China) and heated at 95 °C for 5 min. Sixty micrograms of proteins were loaded on each well of 10% SDS polyacrylamide gel, and then separated at 80 V for 2 h. The gels were equilibrated for 30 min in ice cold transfer buffer, and then the proteins were transferred to PVDF membranes (IPVH00010, Millipore) at 100 V for 2 h at 4 °C. The PVDF membranes were soaked in methanol for 20 s and incubated in ice cold transfer buffer for 5 min. Membranes were blocked with 5% skim milk in TBS-T buffer (20 mM Tris, 137 mM NaCl, 5 mM KCl and 0.05% Tween 20) for 1 h and incubated in the primary antibodies (Table 4) overnight at 4 °C. The PVDF membranes were washed with TBS-T for four times (5 min/time), then incubated with the secondary antibodies (Table 4) for 1 h, which was followed by rinsing in TBS-T buffer for five times. The bands were detected by SuperSignal® West Dura Extended Duration Substrate (34075, Thermo Pierce) and ECL DualVue WB Marker (RPN810, GE). Finally, blots were detected and scanned with an ImageQuant LAS4000 (GE, Stockholm, Sweden). The band intensities were analyzed by the BandScan 5.0 software.

**Table 4:** Antibodies for western blotting.

1^st^ Antibody	Type	Dilution	kDa	
Grx1	Abcam ab45953	1:500	12	
Trx1	Abcam ab26320	1:1500	12	
β-actin	Santa Cruz SC-47778	1:1500	43	
2 ^**nd**^ **Antibody**	**Type**	**Dilution**		
Goat anti-Mouse IgG (H + L) Secondary antibody	Thermo Pierce No: 31160	1:5000		
Goat anti-Rabbit IgG (H + L) Secondary antibody	Thermo Pierce No: 31210	1:5000		

### H & E staining

Muscle samples were fixed in 4% paraformaldehyde at 4 °C for 1 h, and then transferred to room temperature for 12 h. The fixed tissues were dehydrated by ethanol and xylene, and then embedded in paraffin. Cross-sections (7 μm) of fiber were cut and deparaffinized in xylene and then dehydrated in a graded series of ethanol. All sections were stained by hematoxylin-eosin (H & E) then examined by a microscope (BH-2, Olympus, Tokyo, Japan). Each muscle sample had three replicates and six visual fields.

### Immunohistochemistry

After deparaffinization in xylene and dehydration in ethanol, the transverse muscle sections (7 μm) were washed with PBS (8 g NaCl, 0.2 g KCl, 1.44 g K_2_HPO_4_ and 0.24 g KH_2_PO_4_, pH = 7.4) for twice (5 min/time). After incubation with 10 mM citrate buffer (Beyotime, Nantong, China) for antigen unmasking, the sections were incubated in 3% H_2_O_2_ for 10 min to minimize endogenous peroxidase activity^60^, washed in PBS and then blocked in 10% goat serum (Solarbio, Beijing, China) for 10 min at room temperature. The sections were incubated with the primary antibodies of Grx1 (1 μg/ml, ab45953, Abcam, UK) and Trx1 (1:200, ab86255, Abcam, UK) in PBS overnight at 4 °C, and stored at 37 °C for 45 min. The PBS replaced primary antibody as the negative control. After twice rinse in PBS, the sections were incubated by biotinylated goat anti-rabbit antibody (1:1000, ab6721, Abcam, UK) at 37 °C for 30 min. After twice washes in PBS, the sections were treated with DAB Horseradish Peroxidase Color Development Kit (Jiancheng, Nanjing, China) and then treated by hematoxylin. Each slide had 6 visual fields for statistical analysis. The mean OD was used as a parameter for quantification by using ImageJ^61^. Images were transferred gray scale values to OD for each pixel and then a color threshold segmentation approach was implemented for selecting an area of interest. Finally, the mean OD was calculated through integrated OD within the region divided by a measure of the selected area.

### Statistical analyses

All statistical analyses were performed by SAS (version 9.2, Statistics Analysis System). The effects of diet on measured variables were analyzed by ANOVA and means were compared by Duncan’s multiple range test method for multiple comparisons. Statistical significance was set at P < 0.05. Values were shown as means and standard error (SE).
